# Repurposing non-pharmacological interventions for Alzheimer's disease through link prediction on biomedical literature

**DOI:** 10.1038/s41598-024-58604-8

**Published:** 2024-04-15

**Authors:** Yongkang Xiao, Yu Hou, Huixue Zhou, Gayo Diallo, Marcelo Fiszman, Julian Wolfson, Li Zhou, Halil Kilicoglu, You Chen, Chang Su, Hua Xu, William G. Mantyh, Rui Zhang

**Affiliations:** 1 Institute for Health Informatics, University of Minnesota, Minneapolis, MN USA; 2 Division of Computational Health Sciences, Department of Surgery, University of Minnesota, Minneapolis, MN USA; 3 INRIA SISTM, Team AHeaD - INSERM 1219 Bordeaux Population Health, University of Bordeaux, 33000 Bordeaux, France; 4 NITES - Núcleo de Inovação e Tecnologia Em Saúde, Pontifical Catholic University of Rio de Janeiro, Rio de Janeiro, Brazil; 5 Semedy Inc, Needham, MA USA; 6 Division of Biostatistics, School of Public Health, University of Minnesota, Minneapolis, MN USA; 7 Division of General Internal Medicine and Primary Care, Department of Medicine, Brigham and Women’s Hospital, Boston, MA USA; 8 School of Information Sciences, University of Illinois Urbana-Champaign, Champaign, IL USA; 9 Department of Biomedical Informatics, Vanderbilt University Medical Center, Nashville, TN USA; 10 Department of Population Health Sciences, Weill Cornell Medicine, New York, NY USA; 11 Section of Biomedical Informatics and Data Science, School of Medicine, Yale University, New Haven, CT USA; 12 Department of Neurology, University of Minnesota, Minneapolis, MN USA

**Keywords:** Alzheimer's disease, Nutritional supplements, Literature mining

## Abstract

Non-pharmaceutical interventions (NPI) have great potential to improve cognitive function but limited investigation to discover NPI repurposing for Alzheimer's Disease (AD). This is the first study to develop an innovative framework to extract and represent NPI information from biomedical literature in a knowledge graph (KG), and train link prediction models to repurpose novel NPIs for AD prevention. We constructed a comprehensive KG, called ADInt, by extracting NPI information from biomedical literature. We used the previously-created SuppKG and NPI lexicon to identify NPI entities. Four KG embedding models (i.e., TransE, RotatE, DistMult and ComplEX) and two novel graph convolutional network models (i.e., R-GCN and CompGCN) were trained and compared to learn the representation of ADInt. Models were evaluated and compared on two test sets (time slice and clinical trial ground truth) and the best performing model was used to predict novel NPIs for AD. Discovery patterns were applied to generate mechanistic pathways for high scoring candidates. The ADInt has 162,212 nodes and 1,017,284 edges. R-GCN performed best in time slice (MR = 5.2054, Hits@10 = 0.8496) and clinical trial ground truth (MR = 3.4996, Hits@10 = 0.9192) test sets. After evaluation by domain experts, 10 novel dietary supplements and 10 complementary and integrative health were proposed from the score table calculated by R-GCN. Among proposed novel NPIs, we found plausible mechanistic pathways for photodynamic therapy and Choerospondias axillaris to prevent AD, and validated psychotherapy and manual therapy techniques using real-world data analysis. The proposed framework shows potential for discovering new NPIs for AD prevention and understanding their mechanistic pathways.

## Introduction

Alzheimer's disease (AD) and related dementias (ADRD) are chronic and multifactorial neurodegenerative disorders that affect cognition, behavior, functional ability and memory of affected individuals^1^. As of 2020, the worldwide prevalence of ADRD was approximately 50 million, and this number is expected to increase to 152 million by 2050^2^. The high prevalence of ADRD has significant economic, medical, and social consequences for society. In 2019, the global economic burden of ADRD was estimated to be $2.8 trillion, and this burden is projected to increase to $16.9 trillion by 2050^3^. Despite significant advances in our understanding of the etiology and drug targets of AD/ADRD, effective prevention and treatment of these conditions remains elusive. Several medications, including lecanemab^4^ and aducanumab^5^, are thought to reduce the pathological progression of disease processes, but their efficacy is limited and they carry significant side effects^6^. This suggests that our understanding of the pathogenesis of ADRD is incomplete, and novel unbiased approaches are needed to discover new therapies.

AD is a complex and multifactorial disorder that poses significant challenges to drug discovery research. Despite significant progress in this field, there remains an unmet need for effective treatments, prevention, or interventions to slow down the progression of AD^7^. Pharmacological interventions (PI) have demonstrated improvements in cognitive function, albeit with adverse side effects such as nausea, weight loss, leg cramps, and increased mortality risk^8,9^. On the other hand, non-pharmacological interventions (NPI) including sleep^10,11^, diet^12^, dietary supplements (DS)^13^, aerobic exercise^14^, aromatherapy^15^, light therapy^16^ and cognitive training^17^ are widely used by healthcare consumers to enhance their well-being and manage symptoms. Caloric restriction is one of the most well-known methods to prolong healthy life and stave off age-related diseases; emerging evidence suggests that caloric restriction prevents AD in animal models^18^, that aging is associated with a decline in vital nutrients such as taurine^19^, and that supplementation of the deficient nutrient reverses age-associated disease. Thus, NPIs represent a promising, versatile, and potentially cost-effective approach to improve outcomes and quality of life for patients with dementia^20^. Recent studies have demonstrated that certain NPIs may be protective against cognitive decline in individuals with cognitive impairment^21^. For example, aerobic exercise has been shown to benefit various aspects of cognition, including the stabilization of Mini-Mental State Examination (MMSE) scores, as well as improvements in attention, memory, and recognition^22,23^. Cognitive decline may also be attenuated by factors such as improved nutrition, appropriate DS, mental exercise, and social activities^24^. Notably, multimodal NPIs have shown promise in improving cognitive function^25,26^. However, a comprehensive understanding of the effects of NPI, as well as the potential synergistic effects of PI and NPI for AD/ADRD, remains lacking.

Traditionally, new interventions have been developed based on plausible mechanistic hypotheses generated by researchers. However, as the number of potential interventions grows, the intervention discovery and development process faces a bottleneck due to the limits on individual human capacity to evaluate potential hypotheses. In recent years, the computational synthesis of existing data on drugs and diseases has emerged as a promising approach for discovering new therapeutic potentials of existing drugs and identifying treatments for refractory diseases, a practice commonly referred to as drug repurposing^27^. Text mining is a popular data mining approach for drug repurposing due to the rapidly increasing volume of biomedical and pharmaceutical research literature. A vast number of semantic relations between biomedical entities can now be extracted from this literature. Knowledge graphs (KGs), which are heterogeneous networks, can be utilized to store, manage and represent these semantic relations. In biomedical knowledge graphs (BKGs), nodes signify biomedical entities, and edges represent the relationships between two entities^28^. BKGs can provide solutions to practical problems in the biomedical domain. Link prediction (LP) for KGs (also known as KG completion) is the task of inferring missing or potential relations between entities in a KG^29^. The LP for Semantic MEDLINE Database (SemMedDB)^30^ has been found to be effective for drug repurposing for COVID-19^31^.

To address the current lack of research exploring novel NPIs for AD, we first created a comprehensive BKG, named ADInt, encompassing numerous NPIs related to AD. Then we trained and evaluated various LP strategies (e.g., embedding-based, neural network based models) on the ADInt. The best-performing model was further utilized to predict NPIs that may have the potential to prevent AD. The NPIs include natural products (e.g., DS) and complementary and integrative health (CIH). Subsequently, discovery patterns^32^ were employed to generate mechanism pathways for NPI candidates with high scores (i.e., high likelihood), and these pathways are evaluated by domain experts. To further support our findings, we performed real-world data (RWD) analysis to reveal the association between candidate NPIs and ADRD. Our contribution includes creating a novel NPI resource and developing an innovative framework to predict NPIs that may potentially be repurposed for AD prevention. To our best of knowledge, this is the first study to discover NPIs for AD. The developed ADInt and the framework can be applied to NPI discovery for other diseases.

## Methods

**Figure 1:**
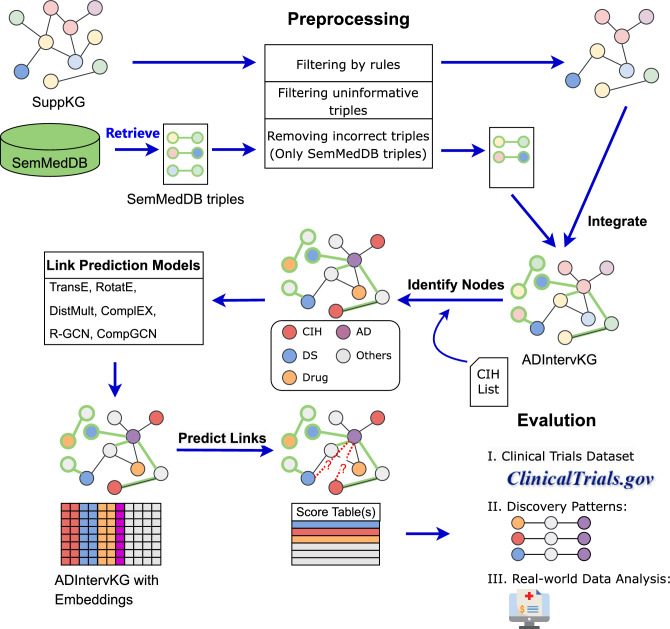
Diagram illustrating the workflow of the methodology.

The complete workflow is depicted in Fig. 1. To investigate the association between NPIs and AD, we initially conducted preprocessing and integration of biomedical triples extracted from SemMedDB and SuppKG^33^. Subsequently, we employed several graph representation models to derive the embedding information of ADInt. Ultimately, we selected the most effective model for generating hypotheses regarding and NPIs for AD and further evaluated them through the discovery patterns and RWD analysis.

### Materials

SemMedDB^30^ is a repository of semantic triples extracted from PubMed abstracts and titles using the SemRep program^34^. We obtained triples from the PREDICTION table of SemMedDB and the source sentences and text of triples from the SENTENCE and PREDICATION_AUX tables. This allowed us to supplement SuppKG with a broader range of information related to interventions for AD beyond the dietary supplement domain. It contains knowledge containing general medicine and related information to ADRD.

Our prior study^33^ found that the current Unified Medical Language System (UMLS)^35^ does not have sufficient coverage of DSs, which is an important category of NPIs. This also limits the representation of supplements in the SemMedDS. Thus, we developed the SuppKG^33^, a KG that focuses on DS. SuppKG comprises 56,635 nodes and 595,222 directed edges, including 2928 DS-specific nodes and 164,738 edges. The nodes in SuppKG are identified by the Concept Unique Identifiers (CUIs) in UMLS, while the predicates in UMLS Semantic Network label the edges. To easily distinguish the DS-specific nodes, a letter "D" was added before the CUI representing the concept of DS. For example, "DC0633482" was used to indicate that "myrtol" (CUI: C0633482) is a DS concept.

SuppKG contains information and triples about DS contained in iDISK^36^ and its extensions, which may not exist in the SemMedDB database. Thus, we integrate SuppKG with SemMedDB to get a comprehensive coverage of DS representation and link to other general medical knowledge.

To validate hypotheses arising from ADInt, we used Electronic Health Record (EHR) data obtained from the University of Minnesota (UMN) Clinical Data Repository E﻿thical approval for this study was obtained from the UMN Institutional Review Board and informed consent was obtained from all subjects and their legal guardians. The cohort under investigation comprised 10,844 individuals who had been diagnosed with mild cognitive impairment (MCI), among whom 978 subsequently received diagnoses of ADRD during the period spanning from 2001 to 2018. Individuals with MCI and ADRD were identified via the International Classification of Diseases (ICD) codes 331.83, 294.9, G31.84, and F09 (for MCI), and 290.40, 290.41, 331.0, 331.11, 331.19, 331.82, G30.0, G30.1, G30.8, G30.9, G31.01, G31.09, G31.83, F01.50, and F01.51 (for ADRD). For the MCI cohort, individuals were required to possess at least one documented diagnosis of MCI and an absence of ADRD diagnoses. The ADRD cohort encompassed individuals meeting the following criteria: (1) receipt of an ADRD diagnosis, (2) documentation of a prior MCI diagnosis preceding the ADRD diagnosis, and (3) a minimum interval of six months between the initial MCI diagnosis and subsequent ADRD diagnosis.

### Preprocessing and integration

To enhance the representation of nodes and relations in the KG, we perform preprocessing before integrating SuppKG and SemMedDB for filtering out generic, uninformative and incorrect triples. The preprocessing includes three steps^31^:

1. *Filtering triples by rules.* First, we removed nodes in the graph that represented generic concepts by referencing the GENERIC_CONCEPT table provided by the SemMedDB database. This table contained concepts such as "Disease" and "Cells," which are known to be too broad to be useful for knowledge discovery. Additionally, concepts with semantic groups that were not likely to be useful for predicting interventions for AD were eliminated, such as "Activities & Behaviors," and "Concepts & Ideas". Finally, only relations that were deemed relevant for LP were kept, including AFFECTS, CAUSES, COEXISTS WITH, PREVENTS, TREATS, etc.
2. *Removing high-degree concepts and uninformative semantic relations.* High-degree concepts in the KG may be too general to be useful for knowledge discovery due to their broad associations with many other concepts. To address this issue, we first computed the out-degree ($k_{i}^{in}$) and in-degree ($k_{i}^{out}$) of each node in the KG. Next, we calculated a log likelihood measure known as $G^{2}$^37^ for each triple, which quantifies the strength of the relationship between the nodes in the triple. The $G^{2}$ formula is given by:
  $$G^{2} = 2\mathop \sum \limits_{i,j,k} n_{ijk} \times log\left( {\frac{{n_{ijk} }}{{m_{ijk} }}} \right), m_{ijk} = \frac{{\mathop \sum \nolimits_{i} n_{jk} \times \mathop \sum \nolimits_{j} n_{ik} \times \mathop \sum \nolimits_{k} n_{ij} }}{{T^{2} }}$$

where $n_{ijk}$ is the item *i, j, k* in the observation table (OT) containing observed frequencies of a triple, $m_{ijk}$ is the item *i, j, k* in the expectation table (ET) describing the expected values assuming independence of terms in triples, and $T = \sum n_{ijk}$. Finally, we normalized $k_{i}^{in}$, $k_{i}^{out}$ and $G^{2}$ and summed them up together to get a final score for each triple. A higher score indicates that the triple is less specific and informative. Consequently, we filtered out some higher-scoring triples to manage the size of the KG to approximately 1.8 M triples, which can be processed by our GPU in a reasonable amount of time.

(3) *Further removing incorrect triples by a trained PubMedBert model.* The triples extracted from the SemMedDB database through SemRep may contain false positives, as the semantics expressed by the triples may differ or be contrary to the content of their source sentences. To address this issue, we utilized a PubMedBert binary classification model that was fine-tuned in our previous work to evaluate the correctness of the triples by referencing their source sentences^31^. The F1 score of this model was 0.854, with a recall of 0.895 and a precision of 0.816.

After preprocessing, we integrated the resulting triples from both sources. For DS concept nodes in SemMedDB triples, we added the letter D before their CUIs to match the identifiers in SuppKG. As the subject and object entities of the integrated triples are identified by UMLS CUIs and their predicates come from the UMLS Semantic Network, we added new triples to SuppKG that did not overlap with its existing triples, without mapping concepts or integrating ontologies. The resulting integrated KG, named ADInt, was obtained.

### NPI nodes identification

We trained and evaluated different approaches to identify nodes representing DS and CIH concepts in ADInt. In SuppKG, DS concept nodes are denoted by a special mark, a letter D added before their CUI. This mark was retained during the integration of SuppKG and SemMedDB triples, allowing us to easily identify these nodes as DS concepts. Unlike DS nodes, nodes describing CIH concepts cannot be identified directly from the KG. To overcome this limitation, we developed a list of CIH concepts, known as the CIH concepts list or CIHLex^38^.

### Link prediction models training and evaluation

A KG can be represented as a labeled directed multi-graph $KG = \left( {E,R,G} \right)$, where *E* denotes the set of nodes representing entities, *R* denotes the set of edges representing relations, and $G \subseteq E \times R \times E$ is a set of triples 〈*h, r, t*〉, where h represents the head entity, *r* represents the relation, and *t* represents the tail entity. Despite the vast amounts of information contained in KGs, they are often incomplete due to various factors, such as noise, missing data, and sparsity. Thus, link prediction (LP) methods seek to infer new triples that may not be explicitly represented in the KG, but which can be logically deduced from the existing ones. The objective of LP aims to predict the most probable entity or relation that completes (*h*, *r*, *?*) (tail prediction), (*h*, *?*, *t*) (edge prediction), or (*?*,* r*, *t*) (head prediction). LP for KGs can be represented as a ranking task, which aims to learn a prediction function that assigns higher scores to true triples and lower scores to false triples. To perform LP on our KG, we explored four KG embedding models (TransE^39^, RotatE^40^, DistMult^41^ and ComplEX^42^) and two graph convolutional network models (R-GCN^43^ and CompGCN^44^).

TransE^39^ is a simple and effective model for LP, particularly for modeling one-to-one relations. In TransE, a triple (*h*, *r*, *t*) is represented as a translation from the embedding of the head entity h to the embedding of the tail entity *t*, with the relation *r* acting as the translation vector in the embedding space. This formulation implies that if a triple (*h*, *r*, *t*) exists, the embedding of entity *h* plus the representation of relation r should be close to the embedding of entity *t*. The TransE score function measures the plausibility of a triple and is defined as follows

$$s\left( {h,r,t} \right) = \left| {\left| {{\varvec{h}} + {\varvec{r}} - {\varvec{t}}} \right|} \right|$$

where ${\varvec{h}}, {\varvec{r}}, {\varvec{t}} \in {\mathbb{R}}^{d}$ is the embedding of *h*, *r* and *t*. Unlike TransE, The RotatE^40^ model converts each relation to a rotation from a head entity to a tail entity in a complex vector space and the score function can be defined as

$$s\left( {h,r,t} \right) = \left| {\left| {{\varvec{h}} \circ {\varvec{r}} - {\varvec{t}}} \right|} \right|$$

where ○ is a Hadamard product.

DistMult^41^ is the most basic semantic matching models, and its scoring function can be defined as

$$s\left( {h,r,t} \right) = {\varvec{h}}^{T} {\varvec{rt}}$$

The drawback of DistMult is that it only works on symmetric relations, that is, the scores of (*h*,*r*,*t*) and (*t*,*r*,*h*) calculated by DistMult are the same. It may cause problems in our KG, for example the triple (Bariatric Surgery, TREATS, Alzheimer's) and the triple (Alzheimer's, TREATS, Bariatric Surgery) should have inconsistent scores. To address this limitation, ComplEX^42^ has been proposed as an extension of DistMult. ComplEX uses a complex vector space and is capable of modeling asymmetric relations. Specifically, head and tail embeddings of the same entity are represented as complex conjugates, which enables (*h*, *r*, *t*) and (*t*, *r*, *h*) to be distinguished. This allows ComplEX to provide consistent scores for both symmetric and asymmetric relations. The scoring function of ComplEX can be defined as follows

$$s\left( {h,r,t} \right) = Re\left( {{\varvec{h}}^{T} {\varvec{rt}}} \right)$$

where *Re* (·) is a real part of a complex vector.

GCNs are a neural network approach for processing graph-structured data^45^. However, most existing GCNs are designed for simple undirected graphs and cannot handle the multiple types of nodes and directed links that exist in our KG. To address this challenge, we explored special graph convolutional neural network models that can handle heterogeneous graphs. Specifically, we evaluated two models: Relational Graph Convolutional Network (R-GCN)^43^ and CompGCN^44^. Based on the architectures of GCNs, R-GCNs consider each different relation and perform feature fusion to participate in updating the hidden states of nodes^43^. The propagation model for calculating the forward-pass update of a node in R-GCNs can be defined as

$${\varvec{x}}_{i}^{{\left( {l + 1} \right)}} = \sigma \left( {\mathop \sum \limits_{{r\epsilon {\mathcal{R}}}} \mathop \sum \limits_{{j\epsilon {\mathcal{N}}_{i}^{r} }} \frac{1}{{c_{i,r} }}{\varvec{W}}_{r}^{\left( l \right)} {\varvec{x}}_{j}^{\left( l \right)} + {\varvec{W}}_{0}^{\left( l \right)} {\varvec{x}}_{i}^{\left( l \right)} } \right),$$

where ${\varvec{x}}_{i}^{\left( l \right)} \ominus \epsilon {\mathbb{R}}^{{d^{\left( l \right)} }}$ is the hidden state of *i*-th nodes in the *l*-th layer of the neural network; ${\mathcal{R}}$ is the set of relations and ${\mathcal{N}}_{i}^{r}$ denotes the neighbor set of *i*-th node under relation $r\epsilon {\mathcal{R}}$; ${\varvec{W}}_{r}^{\left( l \right)}$ and ${\varvec{W}}_{0}^{\left( l \right)}$ are the learnable weight matrix under relation $r$ and self-loop weight matrix in the *l*-th layer respectively; $c_{i,r}$ is a normalization constant that can either be learned or chosen in advance. Using R-GCNs for LP tasks can be regarded as a process of encoding and decoding: an R-GCN producing latent feature vectors of entities and a tensor factorization model exploiting these vectors to predict edges. Taking the DistMult decomposition as an example, the score of a triple (*h*,* r*, *t*) is calculated as^43^

$$s\left( {h,r,t} \right) = {\varvec{h}}^{T} {\varvec{rt}}$$

Thus, to make the model score observable triples higher than negative triples, the loss function can be defined as^43^

$${\mathcal{L}} = - \frac{1}{{\left( {1 + \omega } \right)\left| {\hat{\varepsilon }} \right|}}\mathop \sum \limits_{{\left( {h,r,t,y} \right)\epsilon {\mathcal{T}}}} ylogl\left( {s\left( {h,r,t} \right)} \right) + \left( {1 - y} \right)log\left( {1 - l\left( {s\left( {h,r,t} \right)} \right)} \right)$$

where ${\mathcal{T}}$ is the set of all triples (including positive and negative triples); $\omega$ is the number of negative triples; $\left| {\hat{\varepsilon }} \right|$ is the number of edges; $l$(.) is the logistic sigmoid function; and $y$ is an indicator, where $y = 1$ means triple is positive, otherwise negative.

CompGCN^44^ is another extended version of GCN for heterogeneous graphs, which systematically leverages entity-relation composition operations and jointly learning latent feature vector representations for both nodes and edges in the graph. Different from R-GCNs, CompGCN performs a composition operation Ф over each edge in the neighbor of central node through the embedding of edges and nodes. The update equation of nodes embedding in CompGCN can be defined as^44^

$${\varvec{x}}_{i}^{{\left( {l + 1} \right)}} = f(\mathop \sum \limits_{{\left( {j,k} \right)\epsilon {\mathcal{N}}_{i}^{r} }} {\varvec{W}}_{\lambda \left( k \right)}^{\left( l \right)} \phi ({\varvec{x}}_{j}^{\left( l \right)} ,{\varvec{y}}_{k}^{\left( l \right)} ))$$

where ${\varvec{x}}_{j}^{\left( l \right)}$ and ${\varvec{y}}_{k}^{\left( l \right)}$ are the hidden state of neighboring *j*-th node and its *k*-th relation respectively in the *l*-th layer, and ${\varvec{W}}_{\lambda \left( k \right)}^{\left( l \right)}$ is a relation-type specific parameter, which can be used for direction specific weights. According to whether the edge is the original edge, inverse edge or self-loop edge, ${\varvec{W}}_{\lambda \left( k \right)}^{\left( l \right)}$ will correspond to different weight matrices. $\phi \left( . \right)$ is used to aggregate two vectors of the same size, which can be Subtraction^39^, Multiplication^41^, or Circular-correlation^46^. After updating the node embeddings, we can also update the relation embedding as follows^44^

$${\varvec{y}}_{k}^{{\left( {l + 1} \right)}} = {\varvec{W}}_{rel}^{k} {\varvec{y}}_{k}^{\left( l \right)} ,$$

where ${\varvec{W}}_{rel}^{k}$ is a weight matrix that projects all relations to the same embedding space as nodes, which allows them to be used in the next layer. Similar to R-GCNs LP model, we select a tensor factorization model (convE) to calculate the score of triples. And the same standard binary cross entropy loss function is applied to training the convolutional networks.

The hyperparameters for TransE, RotatE, DistMult, and ComplEX were tuned using a grid search on the validation set for each prediction model. We adjusted the following parameters: learning rate {0.01, 0.001}, number of hidden dimensions {100, 200, 400}, and regularization coefficient {1*10^–6^, 1*10^–9^}. The mini-batch size was set to {250, 1000}. In the case of R-GCN and CompGCN, we conducted tuning on the learning rate {0.01, 0.001}, number of hidden dimensions {100, 200}, number of GCN layers {1, 2}, and maintained a mini-batch size of {250, 500, 1000}. For R-GCN, we applied a dropout layer with a rate of 0.2 to the GCN encoder to prevent overfitting and introduced l2 regularization to the link prediction decoder with a penalty of 0.01. For CompGCN, regularization for the GCN encoder involved a feature dropout rate of 0.1 and a dropout rate of 0.3 after each layer, and the convE decoder employed dropout rates of 0.3 for hidden layer outputs and features. The composition operation employed was circular-correlation. For all models, negative samples were generated by randomly corrupting the heads or tails of positive triples at a 1:20 ratio during the training process.

All work was conducted using Python scripts. The implementation of the TransE, RotatE, DistMult, and ComplEX models was carried out with the DGL-KE 0.1.0.dev0 package^47^ package. Both R-GCN and CompGCN models were constructed using the torch 1.13.1^48^ and DGL 1.0.1^49^ packages. We describe training and evaluation details in the following tasks.

#### Open LBD task

The open discovery approach is specifically aimed at generating innovative hypotheses. Given a head node, the system produces associated tail nodes, thereby facilitating the identification of previously unexplored triple relationships^50^. To evaluate the effectiveness of our LP model, we utilized two evaluation methods.

The first one is Time Slicing^51^. This evaluation approach involves partitioning the KG at a specific time and using the data prior to this time to train the model, and subsequently testing the model on the data following this time to determine if the links formed after the partitioning time can be accurately predicted. Specifically, in our work, we ordered the triples chronologically and divided the KG into training, validation, and testing sets in an 8:1:1 ratio, where the date of publication of the paper mentioning the triple is used as its time, and the partitioning times were set as April 2020 and April 2021, respectively. To evaluate the model performance, we compute ranking-based metrics for each model: mean rank (MR), mean reciprocal rank (MRR), and Hits@k (k = 1, 3, and 10)^39^. Specifically, for each true triple in the testing set, we generated a batch of negative samples by randomly replacing the head or tail nodes while ensuring that these negative samples do not exist in our graph, i.e., we employed corruption with filtering. We then used the trained model to calculate the scores for the true triple and its negative samples, and obtained the ranks of the true triples to calculate the metrics of MR, MRR, and Hits@k. MR represents the average rank assigned to the true triples in the test set:

$$MR = \frac{1}{\left| T \right|}\mathop \sum \limits_{t\epsilon T}^{\left| T \right|} rank\left( t \right)$$

where *T* is all triples in the test set, and *rank(t)* is the position of the triple *t* in the sorted list of t and its negative sample.

MRR is the average inverse rank of all true triples in the test set:

$$MRR = \frac{1}{\left| T \right|}\mathop \sum \limits_{t\epsilon T}^{\left| T \right|} \frac{1}{rank\left( t \right)}$$

Hits@k is the percentage of triples in which the true triple appears in the top k ranked triples:

$$Hits@k = \frac{1}{\left| T \right|}\mathop \sum \limits_{t\epsilon T}^{\left| T \right|} I\left[ {rank\left( t \right) \le k} \right]$$

where *I* is an indicator function. $I\left[ {rank\left( t \right) \le k} \right]$ is equal to 1 if *t* is ranked between 1 and k, 0 otherwise.

In the second evaluation approach, we utilized clinical trial data from ClinicalTrials.gov as a benchmark for predicting potential interventions for AD. Our approach was based on the assumption that interventions under investigation for AD have the potential to be repurposed for other indications. Specifically, we obtained a list of interventions utilized in AD clinical trials registered after April 21, 2020, by conducting a search for the term "Alzheimer" and restricting the results to interventional studies as of November 4, 2022. We excluded control interventions labeled as "placebo," resulting in a total of 671 interventions. We processed these interventions using MetaMap with the UMLS 2022AA release to identify relevant UMLS concepts, resulting in 1606 concepts. The CUIs of these concepts were subsequently used as head nodes, with "PREVENTS" serving as the relations and the "C0002395" (CUI of AD concept) as tail nodes, creating a series of triples for testing. Finally, we employed these newly generated triples based on clinical trial data as another test set to evaluate each trained model.

#### Closed LBD task

The closed discovery method strives to identify the connections between the given head and tail nodes in order to evaluate a specific hypothesis^50^. Although the KG embedding and graph neural network models only provide node and edge representations, patterns from closed discovery were used to infer possible mechanisms for the repurposed interventions. To uncover potential logical connections between concepts in a network, we employed a closed discovery approach by combining sequences of relation types^32^. For DS, The discovery patterns we focused on were:

**InterventionA**-INHIBITS|INTERACTS_WITH-**ConceptB** AND

**ConceptB**-AFFECTS|CAUSES|PREDISPOSES|ASSOCIATED-**Alzheimer’s disease** AND

NOT (**InterventionA**-PREVENTS-**Alzheimer’s disease**)

where InterventionA is a node whose type is DS; ConceptB can be any concept; | indicates logical OR; and for Alzheimer's disease, we focus on the node with CUI C0002395. To analyze the repurposing potential of CIH interventions, we encountered a challenge due to the UMLS semantic types of most CIHs being “topp” (Therapeutic or Preventive Procedure) or “dora” (Daily or Recreational Activity). As these types do not have INHIBIT or INTERACT_WITH relationships to other concepts in the UMLS Semantic Network, and the number of possible paths is not extensive, we did not constrain the predicates in the patterns. The discovery patterns for CIH were:

**InterventionB**—(any predicate)-**ConceptB** AND

ConceptB-(any predicate)-Alzheimer’s disease AND

NOT (**InterventionB**-PREVENTS-**Alzheimer’s disease**

where InterventionB is a node whose type is CIH. We visualized the network structure using ChiPlot (https://www.chiplot.online/).

### Evaluation through RWD analysis

To further support our results, we performed RWD analysis for our predicted non-pharmacological interventions. The DS were identified from the structured medication orders and unstructured clinical notes; and the CIH were identified from the structured Current Procedural Terminology (CPT) codes. Through Power Analysis (see Supplementary Fig. S1 online), we determined that achieving more than 80% statistical power requires a sample size of approximately 1000 individuals, with over 20% of them receiving treatment with either DS or CIH. Upon examination of the dataset, it was revealed that only psychotherapy (42.9%) and manual therapy techniques (28.2%) met this criterion. Subsequently, each 60-day interval following MCI diagnosis was utilized as a time series, extending until the final visit recorded within a ten-year timeframe. Exposure groups for ADRD patients were established based on the utilization of CIHs (Psychotherapy and Manual therapy techniques) by MCI patients. Kaplan–Meier plots were employed to visually represent the unadjusted probability of ADRD within the exposed group. To assess the impact of CIHs on ADRD incidence, a multivariate-adjusted Cox regression model was utilized. The initial model was adjusted for age and sex, while the second model incorporated additional covariates such as delirium, mental retardation, aphasia, depression, anxiety, bipolar disorder, hypertension, hyperlipidemia, vitamin B12 deficiency, and cardiovascular diseases, all of which are known to be associated with ADRD. Furthermore, a case–control dataset was constructed from the MCI patient cohort, with patients eventually diagnosed with ADRD serving as cases. Fisher's exact test was then employed to evaluate statistically significant differences between patients who utilized the predicted DS and those who did not. All analyses were performed using Python 3.9 with the lifelines 0.27, scipy 1.10, and matplotlib 3.7 packages.

### Ethics declarations

All methods were carried out in accordance with the relevant guidelines and regulations.

## Results

### ADInt statistics

ADInt encompasses 162,212 entities across 113 UMLS semantic types, which after further identification include 25,604 Drugs, 16,474 Diseases, 46,060 Genes and Proteins, 2525 DS, and 128 CIH. Furthermore, ADInt comprises 1,017,284 triples, capturing 15 distinct relation types. Detailed statistics can be found in Table 1.

**Table 1:** The frequency and the proportion of relation types in ADInt.

Relations	Counts (%)	Relations	Counts (%)
COEXISTS_WITH	332,428 (32.68)	DISRUPTS	23,238 (2.28)
INTERACTS_WITH	209,448 (20.59)	AUGMENTS	21,912 (2.15)
AFFECTS	96,803 (9.52)	PRODUCES	21,825 (2.15)
TREATS	90,812 (8.93)	PREDISPOSES	13,509 (1.33)
CAUSES	76,235 (7.49)	PREVENTS	12,258 (1.20)
ASSOCIATED_WITH	46,126 (4.53)	COMPLICATES	3519 (0.35)
INHIBITS	39,155 (3.85)	MANIFESTATION_OF	1926 (0.19)
STIMULATES	28,090 (2.76)		
TOTAL	1,017,284 (100.00)		

### Performance of LP models

Table 2 presents the performance obtained by various LP methods using the metrics MR, MRR, and Hits@k^39^. A well-performing model should exhibit a low MR score and high MRR and Hit@k scores. The results demonstrate that the R-GCN model outperforms the other models in all metrics, followed by the TransE and CompGCN model.

**Table 2:** The metrics of link prediction results for different models on integrated KG, ADInt, by time slicing evaluation.

	TransE	RotatE	DistMult	ComplEX	RGCN	CompGCN
Hits@1	0.1770	0.1786	0.1109	0.1062	**0.2656**	0.1520
Hits@3	0.3242	0.3055	0.2586	0.2467	**0.5058**	0.3227
Hits@10	0.5996	0.5340	0.5921	0.5854	**0.8496**	0.6585
MRR	0.3109	0.2987	0.2547	0.2479	**0.4390**	0.3033
MR	8.8607	10.1095	9.2785	9.3799	**5.2054**	7.8198

The best values for each metric are in [bold].

Additionally, Table 3 reports evaluation results of the trained models on the Clinical Trials dataset. The findings show that the R-GCN model performs best in four of the five indicators (Hit@1 is lower than the TransE model and ranks second). In this case, some metrics of the RotatE model (Hits@3, Hits@10 and MR) are better than TransE. Collectively, from both evaluation results presented in Tables 2 and 3, the R-GCN model exhibits the best performance. Thus, we used the R-GCN for further knowledge discovery of NPIs on AD prevention.

**Table 3:** The metrics of link prediction results for different models on integrated KG, ADInt, by clinical trials dataset evaluation.

	TransE	RotatE	DistMult	ComplEX	RGCN	CompGCN
Hits@1	**0.5580**	0.4545	0.2405	0.2143	0.4859	0.3144
Hits@3	0.6294	0.6320	0.3752	0.3058	**0.7071**	0.4152
Hits@10	0.7621	0.8107	0.5391	0.4537	**0.9192**	0.6944
MRR	0.6258	0.5768	0.3543	0.3084	**0.6273**	0.4206
MR	5.4165	5.2284	9.9905	11.5660	**3.4996**	7.6504

The best values for each metric are in [bold].

**Figure 2:**
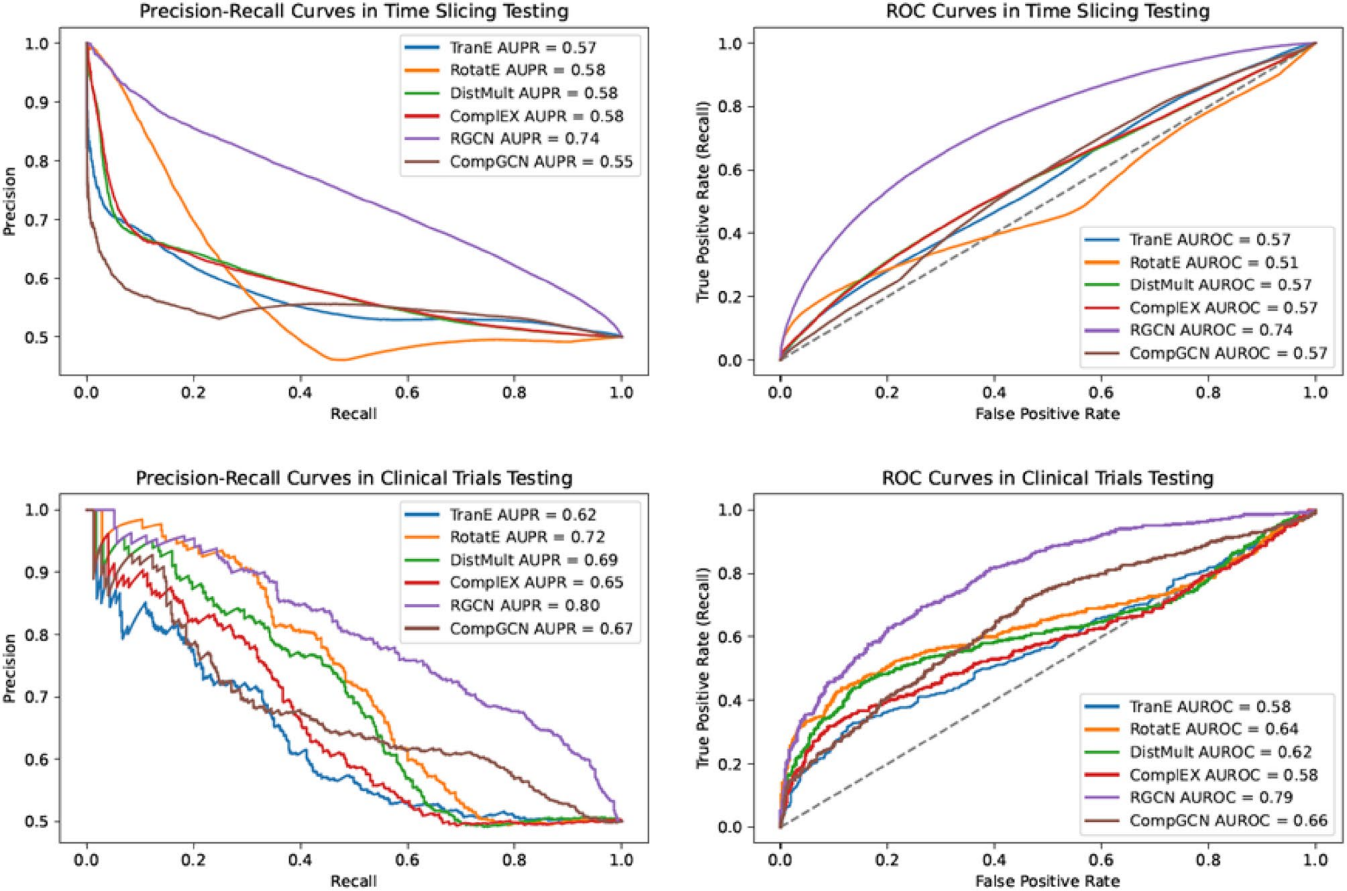
Precision-Recall curves and Receiver Operating Characteristic (ROC) curves of the models in time slicing testing and clinical trials testing.

For open LBD tasks, Fig. 2 displays the Precision-Recall and Receiver Operating Characteristic (ROC) curves for the six models under both time slicing and clinical trials testing conditions. In the time slicing evaluation, the RGCN model stood out by achieving the highest Area Under the ROC Curve (AUROC) of 0.74 and the highest Area Under the Precision-Recall Curve (AUPR) of 0.74. Similarly, in the clinical trials evaluation, it maintained superior performance with the highest AUROC of 0.79 and an AUPR of 0.80.

### Embedding representation of the ADInt KG

**Figure 3:**
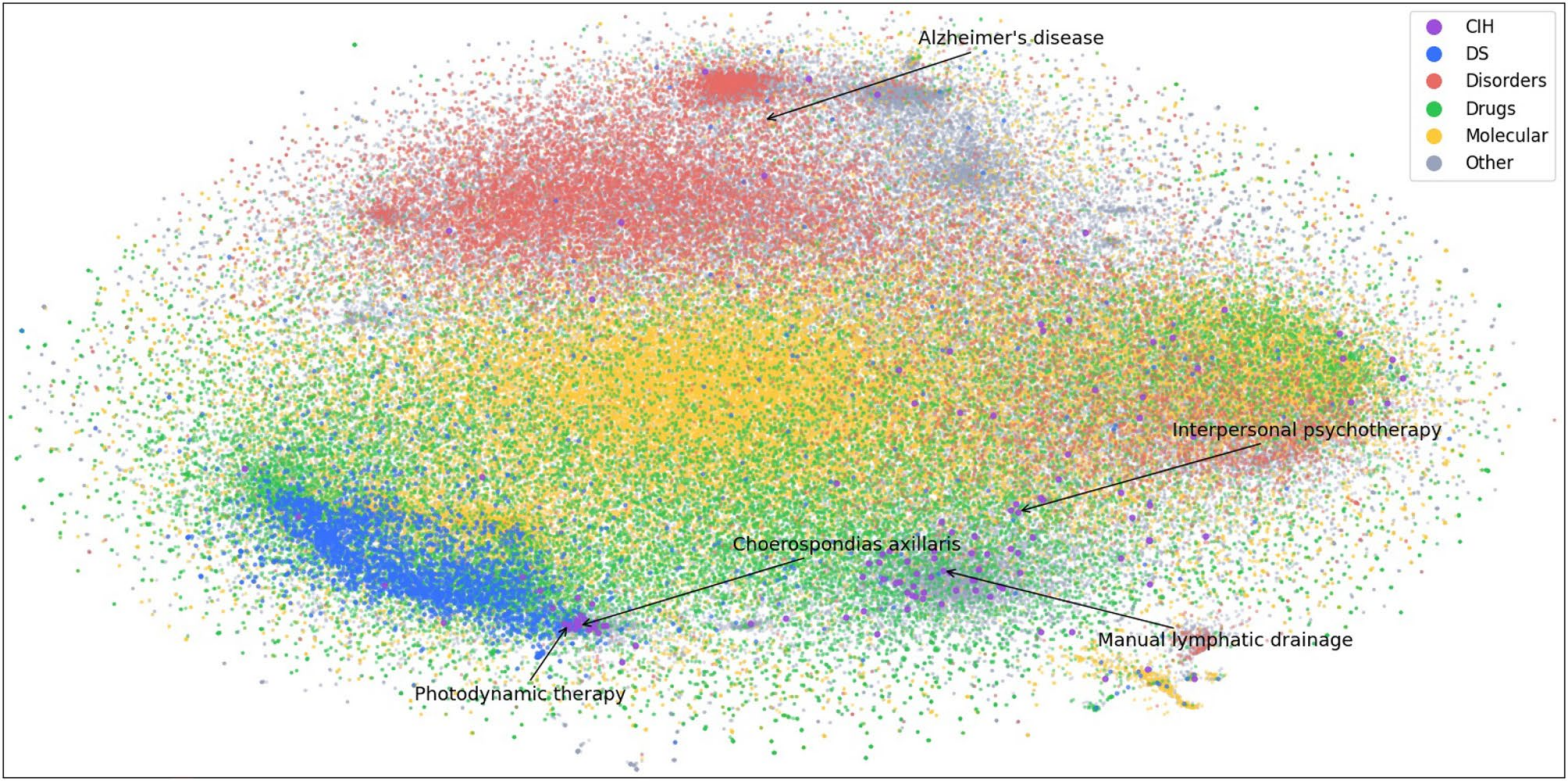
Visualization of nodes in ADInt dimensionally reduced by t-SNE algorithm and shown in a two-dimensional space. Different types of nodes are represented by different colors. Yellow: Molecular. Green: Drugs. Red: Disorders. Blue: DS (dietary supplement). Purple: CIH (complementary and integrative health). Gray: others.

Subsequently, we utilized t-SNE (t-distributed stochastic neighbor embedding)^52^ to obtain the two-dimensional projection of the learned node representations. t-SNE is a technique that reduces high-dimensional data to low-dimensional data while preserving the distribution properties of the original data. Moreover, it expresses the similarity between concepts through the proximity between nodes. As depicted in Fig. 3, nodes with similar types tend to be grouped together, particularly the DS nodes.

### Discovered NPIs for AD prevention

We utilized the embedding information obtained from R-GCN to compute the score of each candidate triple. Specifically, we designated the tail node of these corrupted triples as C0002395 (AD) and the edge as {PREVENTS}. We then attempted to construct different triples by using all NPIs nodes in the graph as head nodes and calculated their score using the R-GCN model. Our focus was solely on the discovery of novel triples; thus, we excluded triples that already existed in ADInt. For novel triples, a higher score indicated a higher probability of being closely related to the true relationship. We categorized the triples into two groups based on the type of the head node, including DS and CIH, to discover novel NPIs for AD. After evaluation by experts, the top 10 predicted novel candidates for AD are presented in Table 4.

**Table 4:** Top 10 proposed entities for different categories with predicate PREVENTS.

	Dietary Supplement (DS)	Probability(s) for DS	Complementary and Integrated Health (CIH)	Probability(s) for CIH
1	Desmodii herba	0.9759	Mindfulness relaxation	1.0000
2	Tamaris	0.7372	Massage therapy	0.7309
3	Glucomannan	0.6030	Cold therapy	0.4104
4	bidens pilosa	0.4859	Interpersonal psychotherapy	0.4062
5	Lutein	0.4819	Photodynamic therapy	0.3790
6	Millet (as grain, fiber)	0.4699	Myofascial release	0.2458
7	Artichoke	0.4641	Guided imagery	0.2455
8	Damask rose	0.4303	Art therapy	0.2210
9	Caryophyllus aromaticus	0.3942	Manual lymphatic drainage	0.1751
10	Shark liver oil	0.3060	Laughter therapy	0.1017

**Figure 4:**
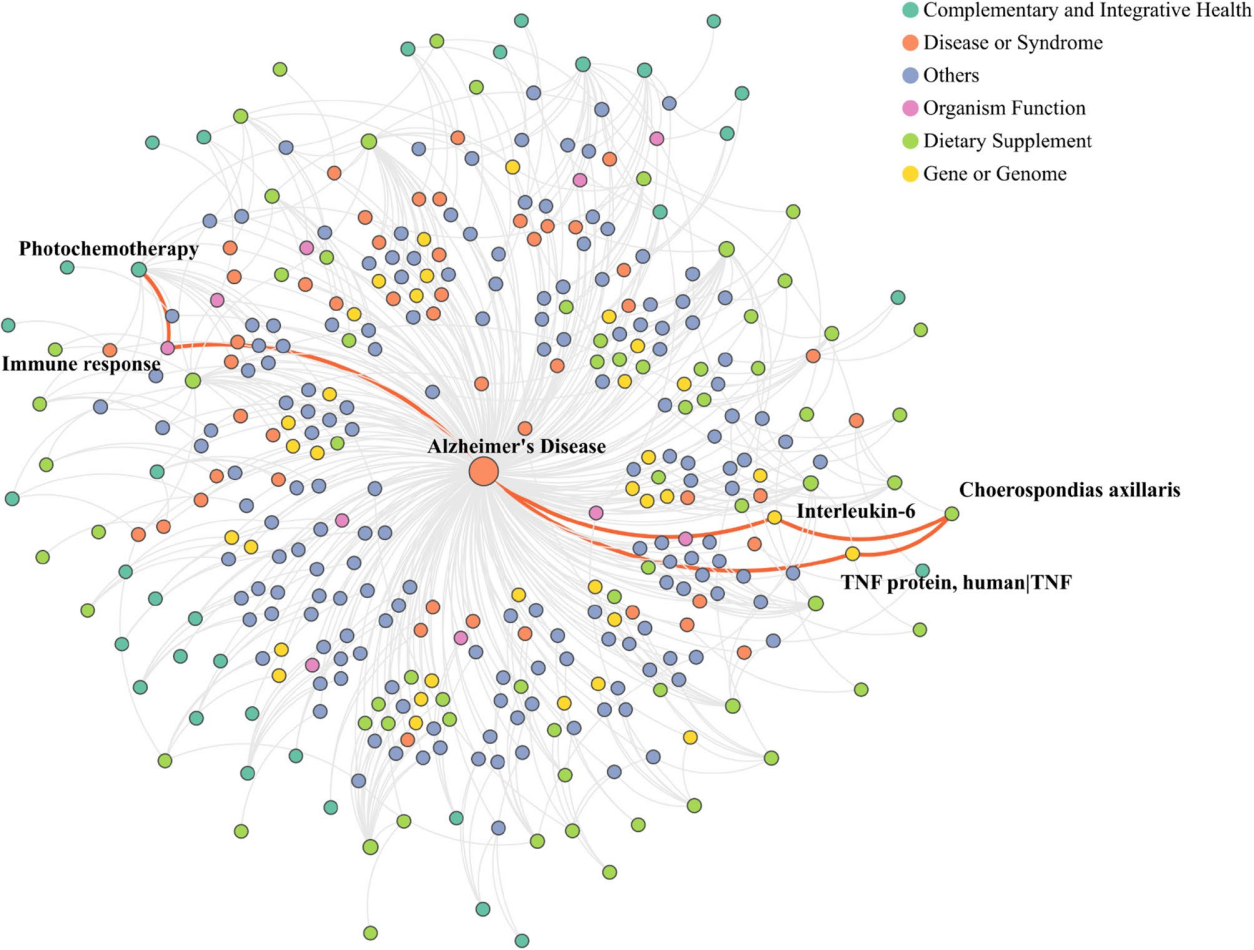
Top-ranked predicted results of ADInt-based exploration.

Figure 4 displays the network structure of the top-ranked predicted results. The network highlights three pathways that include a set of interesting findings, which will be further discussed in the following sections. Specifically, this pathway reveals potential mechanisms through which CIH and DS may influence the risk of AD, and suggests potential targets for therapeutic interventions. The identified associations and pathways represent a promising direction for future research into the prevention of AD.

### Evaluation of novel NPIs for AD prevention through RWD analysis

**Figure 5:**
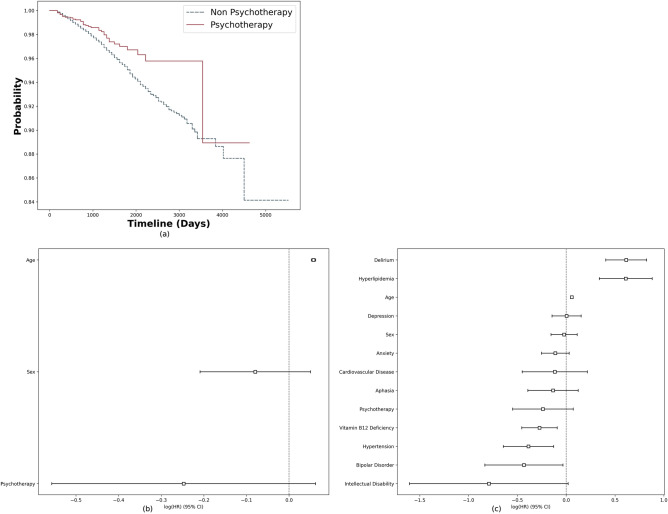
RWD analysis results for Psychotherapy: (**a**) The Kaplan–Meier survival plots for the MCI cohort, (**b**) multivariate-adjusted Cox regression model for ADRD (adjusted for age and sex), (**c**) multivariate-adjusted Cox regression model for ADRD (adjusted for age, sex and disease likely to cause ADRD).

The study cohort comprised 10,844 patients diagnosed with MCI, among whom 978 were subsequently diagnosed with ADRD. Among the 978 patients with ADRD, 276 reported receiving Psychotherapy, while 875 MCI patients without ADRD reported the same intervention. Fisher's exact test was employed to examine the potential association between Psychotherapy and ADRD diagnosis, revealing a statistically significant difference in Psychotherapy usage between the ADRD and non-ADRD groups (*p*-value < 0.001). Figure 5a presents Kaplan–Meier survival plots for the MCI cohort, illustrating the probability of non-progression from MCI to ADRD over time. Noticeable discrepancies between the curves suggest that increased involvement in Psychotherapy may correlate with reduced risk of ADRD development (*p*-value = 0.007). Upon adjustment for age and sex in the multivariate analysis, individuals who received psychotherapy had a lower rate of progression to ADRD compared to those who did not (Hazard Ratio (HR) 0.78, 95% Confidence Interval (CI) 0.57–1.06) (Fig. 5b). This association persisted even after controlling for various comorbidities associated with ADRD (HR 0.79, 95% CI 0.58–1.07). However, it did not reach statistical significance at the conventional 0.05 level. The comorbidities include delirium, intellectual disability, aphasia, depression, anxiety, bipolar disorder, hypertension, hyperlipidemia, vitamin B12 deficiency, and cardiovascular disease (Fig. 5c).

**Figure 6:**
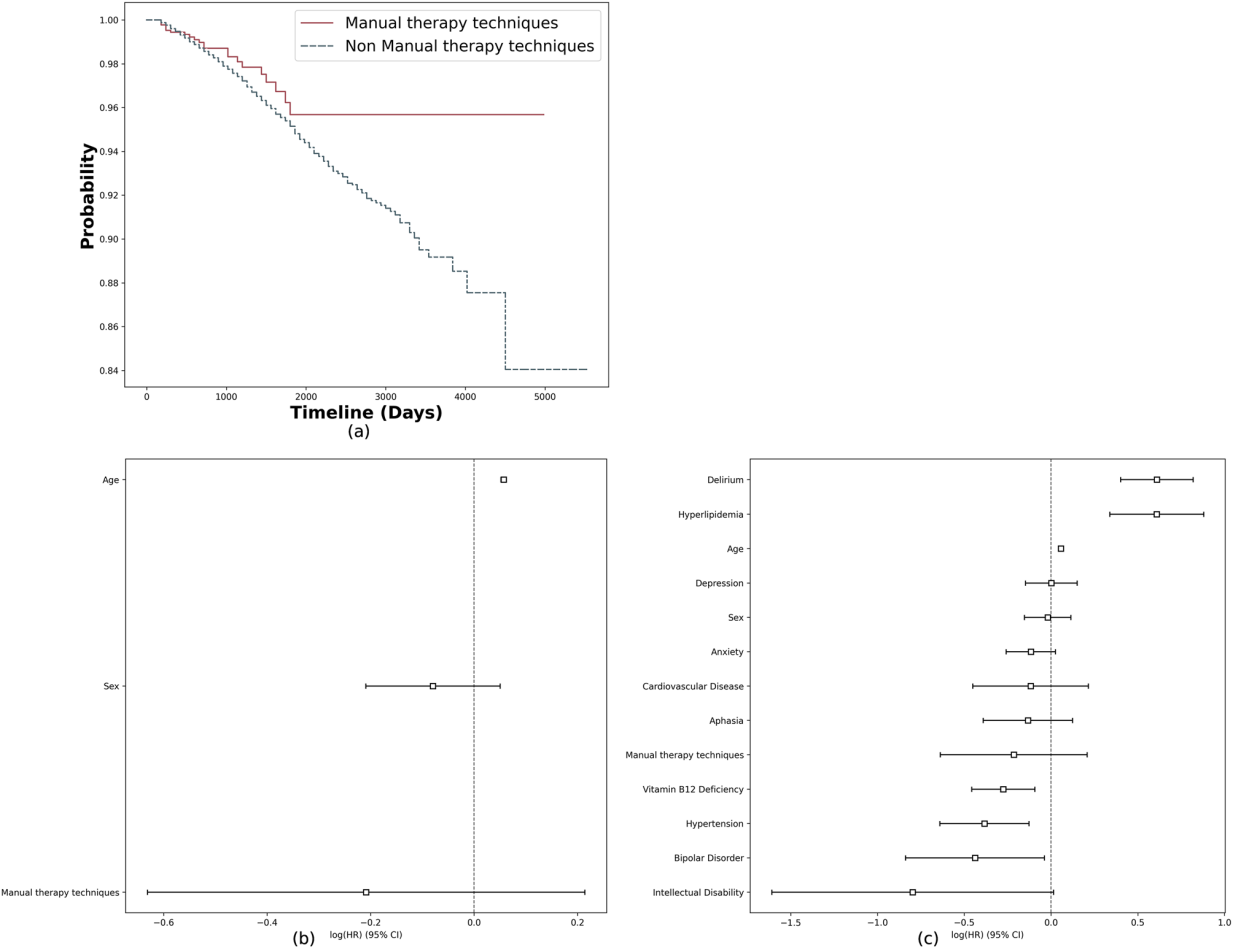
RWD analysis results for manual therapy techniques: (**a**) The Kaplan–Meier survival plots for the MCI cohort, (**b**) multivariate-adjusted Cox regression model for ADRD (adjusted for age and sex), (**c**) multivariate-adjusted Cox regression model for ADRD (adjusted for age, sex and disease likely to cause ADRD).

Additionally, among the 978 patients diagnosed with ADRD, 411 reported receiving Manual therapy techniques, while 1402 MCI patients without ADRD reported the same intervention. Fisher's exact test demonstrated a statistically significant difference in the utilization of Manual therapy techniques between the ADRD and non-ADRD groups (*p* < 0.001). Kaplan–Meier survival analysis suggested that increased involvement in manual therapy techniques may be linked to reduced risk of ADRD development (*p*-value = 0.1) (Fig. 6a). After adjusting for age and sex in the multivariate analysis, individuals who received Manual therapy techniques had a lower rate of progression to ADRD compared to those who did not (HR 0.81, 95% CI 0.53–1.24) (Fig. 6b). This association remained robust after accounting for various comorbidities associated with ADRD (HR 0.81, 95% CI 0.53–1.23), but was not statistically significant at the 0.05 level (Fig. 6c).

## Discussion

In this study, we trained and compared various LP methods on the task of knowledge discovery. The R-GCN model has demonstrated superior performance over other models on both the time slicing and clinical trials test sets (see Tables 2, 3). Notably, TransE exhibited the second-best overall performance in ranking-based metrics, which is consistent with our prior work^31^ demonstrating that relatively simple TransE outperformed other KG embedding methods (RotatE, DistMult, ComplEX) on the extended SemMedDB. We speculate that the poor performance of DistMult and ComplEX is due to their preference for high-degree entities, which we removed during the data preprocessing stage^53^. We believe that the reason for RotatE's underperformance is similar, as our filtered KG emphasizes simple relations. Although RotatE addresses some of the limitations of TransE in handling multiple and symmetric relations by introducing complex spaces^40^, our findings suggest that this approach may not be appropriate for our KG. The superior performance of R-GCN suggests that the neighborhood aggregation operation of the graph convolution network is useful for learning graph representations^54^. However, we found that another graph convolutional network-based model, CompGCN, had a mediocre performance. We hypothesize that CompGCN's reliance on linear transformations for relation embeddings does not suit our KG^55^. Additionally, our evaluation of R-GCN on the Clinical Trials dataset outperformed its performance on the time slicing evaluation. These results demonstrate that R-GCN is adept at distinguishing which subjects are feasible for preventing AD. It is worth noting that while our experiments confirm R-GCN as the optimal LP model, metrics such as MR, MRR, and Hits@k only reflect the model's ability to predict interventions being trialed or known interventions. Indeed, models with low metrics may still produce valuable results^31^. Nevertheless, these metrics can inform model selection for NPI repurposing.

We used discovery patterns to generate mechanistic pathways for high-scoring triples predicted by the R-GCN model through the Neo4J platform. Photodynamic therapy (PDT) is a clinically used approach for treating or preventing various medical conditions, ranging from age-related macular degeneration to malignant tumors such as prostate cancer patients. PDT involves the use of light and a photosensitizing chemical substance along with molecular oxygen to elicit cell death^56^. Recently, PDT has been proposed as a potential therapeutic option for AD^56^. The precise mechanism of how PDT can provide therapeutic benefits for AD remains elusive, and the practical use of PDT for treatment or prevention of AD is basically non-existent given that tissue must be directly exposed to light, which is not feasible when dealing with the entire brain. However, this finding provides theoretical support for treating AD through modulation of the immune system. For instance, a study evaluating the use of PDT with 5-aminolevulinic acid on mice has reported that it affects the immune response^57^. The study found that there was a significant reduction in the mRNA expression of interleukin-22 (IL-22), a cytokine produced by several immune cells that is associated with inflammation. Converging evidence has demonstrated that immune/inflammation response plays a crucial role in the initiation and regulation of AD^58^. Thus, our PDT finding, while based on a therapy that has major practical limitations for treating AD, highlights immune mechanisms for preventing and treating AD. It should be noted that this is a preliminary finding based on a limited number of studies, and more research is needed to confirm these results.

Choerospondias axillaris, commonly known as Nepali hog plum, is a fruit that is approximately three centimeters long with sour flesh and yellow skin. Plums and other yellow-skinned fruits, such as papayas, tangerines, and oranges, are high in ß-cryptoxanthin, an antioxidant. A recent study^59^ found an inverse association between serum β-cryptoxanthin levels and the incidence of AD and all-cause dementias in individuals who consumed yellow-skinned fruits. Specifically, an increase of 8.6 micromole/liter in serum β-cryptoxanthin levels was associated with a 14% decreased risk of AD. To propose a potential mechanism for this protection, we examined the patterns between Choerospondias axillaris and AD. In a study^60^, it was found that Choerospondias axillaris inhibits both TNF protein and interleukin-6. These two inflammation mediators are well-known inducers of AD, as demonstrated in previous studies^61,62^. Specifically, interleukin-6 has been linked to the pathogenesis of AD, while tumor necrosis factor-α has been proposed as a potent therapeutic target for AD. Lutein, a carotenoid also found in Choerospondias axillas, is also found as a protective intervention. This finding corroborates prior reports that demonstrated an inverse association between lutein intake and dementia occurrence^62^. Furthermore, increased lutein intake has been associated with lower levels of AD neuropathology postmortem^63^. Overall, Choerospondias axillaris and other yellow-pigmented fruits may act as protectors by reducing the levels of pro-inflammatory cytokines crucially implicated in AD. Finally, it is interesting to note that some of our findings (from Table 4) may have clinical impact in the prevention of AD and have not been published as such in the biomedical literature. For example, glucomannan and millet are dietary fibers (prebiotics) that modulate the gut microbiome, which has been discussed to have beneficial effects in the prevention of cognitive decline^64^. Some other interventions in Table 4 have not been discussed at all as preventive for AD (i.e. Interpersonal psychotherapy, mindfulness relaxation, and myofascial release), but are of clinical relevance. Interpersonal psychotherapy and manual lymphatic drainage, as identified in our predicted results, fall under the categories of psychotherapy and manual therapy techniques, respectively, and RWD analysis revealed their potential to reduce the risk of developing ADRD. The results from the Kaplan–Meier survival analysis indicate that both psychotherapy and manual therapy techniques may contribute to lowering the risk of ADRD. However, these findings did not reach statistical significance, suggesting the need for additional studies to more conclusively determine their effects. Furthermore, the observational nature of our RWD does not allow us to rule out the possibility that the observed differences between groups receiving and not receiving particular interventions is due to unmeasured confounding.  Constrained by the capacity of the local EHR, a power analysis and review of the dataset revealed that only the sample sizes for psychotherapy and manual therapy techniques met the requirements for sufficient statistical power. Thus, in the future studies, we will leverage multi-site larger EHR data to examine and analyze other NPIs, potentially gaining broader insights into ADRD prevention.

There are several possibilities for future improvements to our approach. Firstly, we augmented SuppKG with triples extracted from the SemMedDB database, indicating that all triples in our ADInt were obtained through literature-based discovery. In order to further enhance our knowledge graph, we can merge it with other comprehensive biomedical databases and biological networks, such as DrugBank and KEGG^65^. This will enable us to expand the scope of our analysis and identify additional relevant interventions. Secondly, in addition to knowledge graph embedding and graph neural network models, other methods such as link prediction based on language models have also demonstrated promising results on LP tasks. These methods could also be explored in future studies on drug repurposing. Lastly, since the determination of the plausibility of an intervention and its pathways to AD is a labor-intensive process, only the top 10 of each scoring table were evaluated by experts. However, in future work, larger samples could be considered if the necessary resources are available.

## Conclusions

Our analysis emphasizes the growing importance and popularity of studying NPIs in the context of disease management. By demonstrating the efficacy of our approach in revealing intricate relationships between biomedical entities, particularly NPI entities, and diseases of interest, we provide plausible mechanistic explanations for these associations. Notably, our contributions in this field include creating valuable NPI resources and developing an innovative framework to predict NPIs that may potentially be repurposed for AD. To the best of our knowledge, this is the first study that specifically aims to discover NPIs for AD. Furthermore, the versatility and adaptability of our approach enable its application to NPI discovery for a wide range of other diseases. Our proposed approach also holds significant potential in addressing various clinical questions, such as the discovery of drug adverse reactions and drug-drug interactions, further emphasizing the importance and applicability of our research in the broader biomedical field.

### Supplementary Information

Supplementary Information.

## Data Availability

ADInt knowledge graph data is available in the following google drive: https://github.com/zhang-informatics/ADInt. The complete SemMedDB database can be accessed directly on https://lhncbc.nlm.nih.gov/ii/tools/SemRep_SemMedDB_SKR.html.

## Code availability

The code used for data preprocessing, model training, result evaluation and visualization in this study is available in the following repositories: https://github.com/YKXia0/LBD_AD.
