# Supplementary material for: Repurposing non-pharmacological interventions for Alzheimer's disease through link prediction on biomedical literature
Source: Sci Rep. 2024 Apr 15;14:8693. doi: 10.1038/s41598-024-58604-8 (PMC11018822; doi:10.1038/s41598-024-58604-8)
Supplement: Supplementary file 1 — Supplementary Information. [file 41598_2024_58604_MOESM1_ESM.pdf]

Repurposing Non-pharmacological Interventions for Alzheimer's Disease through Link Prediction on Biomedical Literature

Yongkang Xiao, MS[1,#], Yu Hou, PhD[2,#], Huixue Zhou, BM[1], Gayo Diallo, PhD[3], Marcelo Fiszman, MD, PhD[4,5], Julian Wolfson, PhD[6], Li Zhou, MD, PhD[7], Halil Kilicoglu, PhD[8], You Chen, PhD[9], Chang Su, PhD[10], Hua Xu, PhD[11], William G. Mantyh, MD[12], Rui Zhang, PhD[2,*]

[1]Institute for Health Informatics, University of Minnesota, Minneapolis, MN, USA
[2]Department of Surgery, University of Minnesota, Minneapolis, MN, USA
[3]INRIA SISTM, Team AHeaD - INSERM 1219 Bordeaux Population Health, University of Bordeaux, F-33000, France
[4]NITES - Núcleo de Inovação e Tecnologia Em Saúde, Pontifical Catholic University of Rio de Janeiro, Brazil
[5]Semedy Inc, Needham, Massachusetts, USA
[6]Division of Biostatistics, School of Public Health, University of Minnesota, Minneapolis, MN, USA
[7]Division of General Internal Medicine and Primary Care, Department of Medicine, Brigham and Women's Hospital, Boston, MA, USA
[8]School of Information Sciences, University of Illinois Urbana-Champaign, Champaign, IL, USA
[9]Department of Biomedical Informatics, Vanderbilt University Medical Center, Nashville, TN, USA
[10]Department of Health Service Administration and Policy, Temple University, Philadelphia, PA, USA
[11]Section of Biomedical Informatics and Data Science, Yale University, New Haven, Connecticut, USA
[12]Department of Neurology, University of Minnesota, Minneapolis, MN, USA

# Authors contributed equally

* Corresponding author:
Rui Zhang, PhD
University of Minnesota
zhan1386@umn.edu

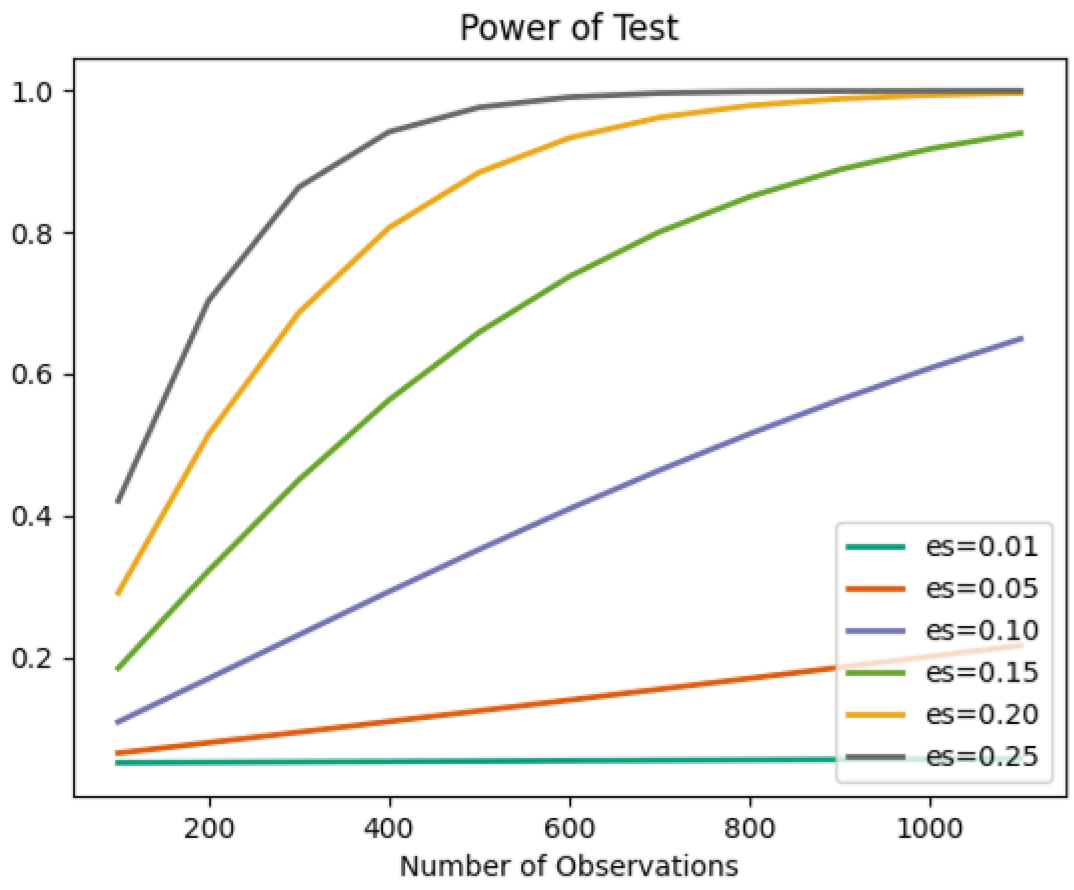

Supplementary Figure S1: Results of Power Analysis (es: effect size, the quantified magnitude of a result present in the population)
